# Improving the Quality and Value-Added Potential of Crude Pangasius Oil Extracted From Belly Byproducts via Rendering Methods

**DOI:** 10.1155/sci5/7188982

**Published:** 2025-09-30

**Authors:** Nami Lestari, Lukman Junaidi, Armen Zulham, Abdullah bin Arif, Tri Heru Prihadi, Mulyana Hadipernata, Suroto Hadi Saputra, Irin Iriana Kusmini, Muhammad Syukur Sarfat, Ermiati, Eddy Sapto Hartanto, Bedy Sudjarmoko, Angela Mariana Lusiastuti, Mulyasari, Sri Turni Hartati

**Affiliations:** ^1^Research Center for Agroindustry, Research Organization for Agriculture and Food, National Research and Innovation Agency (BRIN), Tangerang Selatan, Banten, Indonesia; ^2^School of Postgraduate Studies, Universitas Djuanda, Bogor, West Java, Indonesia; ^3^Research Center for Cooperative, Corporation, and Peoples Economy, Research Organization for Governance, Economy, and Community Welfare, National Research and Innovation Agency (BRIN), Jakarta, Indonesia; ^4^Research Center for Fishery, Research Organization for Earth Sciences and Maritime, National Research and Innovation Agency (BRIN), Cibinong, West Java, Indonesia; ^5^Regional Research and Development Agency of East Kalimantan Province, Samarinda, East Kalimantan, Indonesia; ^6^Research Center for Applied Zoology, Research Organization for Life Sciences and Environment, National Research and Innovation Agency (BRIN), Cibinong, West Java, Indonesia; ^7^Engineering Department, Faculty of Agricultural Technology, IPB University, Jl. Raya Dramaga, Kampus IPB Dramaga, Bogor, West Java, Indonesia; ^8^Research Center for Veterinary Science, Research Organization for Health-National Research and Innovation Agency (BRIN), Cibinong, West Java, Indonesia; ^9^Research Center for Marine and Land Bioindustry, Research Organization for Earth Sciences and Maritime, National Research and Innovation Agency (BRIN), North Lombok, West Nusa Tenggara, Indonesia; ^10^Research Center for Conservation of Marine and Inland Water Resources, National Research and Innovation Agency (BRIN), Cibinong, West Java, Indonesia

**Keywords:** belly fish, dry rendering, pangasius fish fillet, trimming meat, wet rendering

## Abstract

The high demand for Pangasius fillets in Indonesia has resulted in substantial byproduct waste, contributing to environmental concerns. To overcome this problem, it is necessary to utilize waste to create economically viable products, such as Pangasius fish byproduct oil. This study aimed to evaluate the quality of crude Pangasius oil extracted from fillet processing byproducts (belly and trimming) using dry and wet rendering techniques and to assess its business potential with a value-added approach using the Hayami method. The oil extraction methods used include dry and wet rendering. The study examined two main treatment categories: extraction method (A), which included dry rendering (A1) and wet rendering (A2), and the types of Pangasius fillet industry byproducts (B), which comprised trimming (B1) and belly (B2). The best oil, extracted from the belly via dry rendering, showed favorable physicochemical properties: free fatty acids (0.88%), peroxide value (5.85 mEq/100 g), iodine value (65.55 g I_2_/100 g), and saponification value (211.84 mg KOH/g). The oil was found to have considerable quantities of vitamins A (161.65 IU/g), D (192.40 IU/g), and K (3.20 IU/g), along with elevated levels of palmitic (40.70%), oleic (21.20%), and linoleic acid (10.60%). An economic assessment indicated that 1 kg of byproducts could produce 0.25 L of crude oil, which has a value-added of U.S.$ 1.62/L. These results emphasize the potential of Pangasius belly oil as a value-added product, offering promising functional and commercial opportunities in the food and nutraceutical sectors. by dry extraction has the potential to be developed into a future commercial food product.

## 1. Introduction

Aquaculture is one of the businesses in the fisheries sector that is developing in Indonesia. This business has the potential to support one of the sustainable development goals of the Ministry of Maritime Affairs and Fisheries, Indonesia, namely, increasing the production and productivity of marine and fishery businesses. This development is expected to boost the Indonesian economy, create job opportunities, and provide supplementary income sources. One of the most commonly cultivated and favored fish among Indonesians is Pangasius (*Pangasius* spp.) [1, 2]. Pangasius fish is a fishery commodity with relatively high production levels in Indonesia, which continue to increase annually [2, 3]. The Director-General of Marine Competitiveness and Fisheries of the Ministry of Marine Affairs and Fisheries, Indonesia, has dismissed the idea that Pangasius fish are not in great demand in the world market. In contrast, Pangasius fish is one of the most sought-after fish in the global market, with a projected Indonesian production of 380,000 tons by 2022, most of which is absorbed by the domestic market. The high demand for Pangasius fish in Indonesia is, of course, supported by the demand for the fillet Pangasius industry, but this will have an impact on the increasing number of byproducts produced by the industry. This is according to what was reported by several researchers, where the processing of Pangasius fish in Indonesia produces Pangasius fillets, which are then sold in the form of fresh or frozen Pangasius fillets [2, 4]. The yield from the processing of Pangasius fillets is only approximately 45%, resulting in a relatively large percentage of waste.

To date, most of Pangasius fish production has been processed into Pangasius fish fillets intended as an export commodity. Suppose that the production of Pangasius fillets reaches 400 tons per month or 1200 tons of whole Pangasius fish. In this case, there is significant potential for extracting crude oil from byproducts. The meat yield that can be utilized in processing Pangasius fish is only 49%, with the remaining 51% being waste from the head, skin, offal, and bone [5]. Assuming a 45% oil yield from 612 tons of viscera (51% of 1200 tons), the potential crude fish oil production would be approximately 275 tons per month [6].

The presence of these byproducts can undoubtedly harm the environment if not handled properly. Therefore, developing product diversification based on byproducts from the Pangasius fillet industry is necessary for implementing a clean production system. It is hoped that these byproducts can provide food and nutrition and increase value-added and community income [2]. Various alternatives have been implemented to utilize these byproducts, including their use as raw materials for byproduct-based fish oil extraction [2, 7]. Several studies have reported that 1 kg of Pangasius fish byproduct (belly flap, offal, head, tail, and fins) produced 116 mL of crude Pangasius oil [8]. The potential of Pangasius byproducts, including offal, head, tail, and fins, is significant for various applications [8]. Among these byproducts, trimmings and belly tissues were specifically chosen for this study due to their relatively high lipid content and accessibility during processing. Trimming portions are composed of skin, subcutaneous fat, and muscle residues, while belly flaps contain visceral-adjacent adipose tissues. Pangasius offal, consisting of the stomach, intestines, liver, and other internal organs, can be processed into animal feed due to its high protein and fat content [9]. Fish protein hydrolyzate (HPI), a liquid product made from fish with proteolytic enzymes, can also be derived from Pangasius offal and used as a dietary supplement or food to increase protein intake [10]. The head, tail, and fins, along with other trimmings, can be utilized to produce fish oil rich in omega-3 fatty acids [8]. Fish oil has several health benefits, including preventing heart rhythm disorders and lowering blood pressure [9]. Additionally, these parts can be processed into HPI with calcium-binding activity. This shows that there is still potential to utilize byproducts of processing catfish fillets into products of economic value, namely fish oil.

The yield level of crude oil obtained from Pangasius fillet industry byproducts is, of course, influenced by the extraction method. The extraction was carried out using a dry rendering system [7, 10], a wet rendering system [8], and extraction using solvents [11, 12], with dry rendering and solvent extraction being the most prominent. Recent advancements in fish oil extraction have highlighted the potential of rendering methods to optimize yield and quality, yet critical gaps remain in understanding their comparative efficacy for pangasius viscera. While wet rendering is widely adopted for its cost-effectiveness and high polyunsaturated fatty acid (PUFA) retention (44% in tuna heads) [13], studies reveal persistent challenges, including oxidative instability (peroxide values [PVs] up to 7.26 meq/kg at 100°C) [14] and impurities from residual proteins [13, 15]. Conversely, dry rendering demonstrates superior oxidative stability at optimal temperatures (100°C, showing 15.82 iodine value (IV) vs. 16.01% in wet methods) [14, 16], but its yield remains suboptimal compared to solvent-based techniques [17].

These various methods have their respective advantages and disadvantages. However, what is of interest in this research is how the extraction method used can be safe for the environment (minimal waste) and can be used by small and medium businesses. Therefore, this research uses two extraction methods that are considered to have minimal waste and are effectively used by small and medium businesses, namely wet and dry rendering systems. The hypothesis posits that integrating dry rendering's thermal efficiency with wet rendering's PUFA preservation could address these dual limitations, particularly for pangasius belly, an underutilized byproduct constituting 10%–12% of fish weight [15]. This approach aims to bridge the research gap identified in recent studies [13, 18] that primarily focus on single-method optimizations rather than synergistic combinations, potentially enhancing both the economic value and nutritional profile of crude pangasius oil through improved extraction efficiency (targeting > 12.4 g/100 g yield) and oxidative stability [15].

To evaluate the feasibility of the Pangasius fish byproduct oil extraction industry, it is necessary to calculate the added value obtained, using the Hayami method. This method is beneficial for analyzing the economic value of various industries by mapping actors, volume, and value in the supply chain. The Hayami method can be modified to suit specific business needs, such as the number of organizations in a chain and multi-year continuous business cycles. It has been applied in various studies to evaluate value added and income [19]. The Hayami method is a widely used approach for analyzing value-added in agro-industrial products, including fishery products [20, 21]. According to previous research results, the use of the Hayami method facilitates the identification of the amount of value-added and profitability at each stage of fishery product processing, where the positive value-added indicates that processing efforts provide economic contributions to both business actors and workers [21–23]. In addition, previous research results confirm that diversification and increased efficiency of the production process greatly affect the amount of value-added generated in fishery product processing.

The novelty of extracting crude Pangasius oil from byproducts lies in its ability to transform waste into a valuable product, its high oil yield, the exploration of various extraction methods, compliance with health standards, and contribution to environmental sustainability. This approach enhances the economic viability of the Pangasius fish industry and supports health and wellness initiatives. The process focuses on utilizing fish byproducts, such as bellies and trimmings, which are often discarded in fillet production. This reduces waste and adds economic value to parts of the fish that are typically considered to have low value. This extraction process contributes to more sustainable practices within the fish-processing industry by converting fish-processing waste into valuable oil. It addresses the environmental concerns related to waste disposal and promotes a circular economy. Therefore, this study aimed to identify the characteristics of crude Pangasius oil from the industrial byproducts of Pangasius fillets (trimming and belly) produced using wet and dry rendering methods.

## 2. Materials and Methods

### 2.1. Materials

The materials used in this research were industrial byproducts of Pangasius fillets (trimming and belly) obtained from small and medium fillet industries in the Tulungagung and Pasuruan areas, East Java, Indonesia. Plastic bottles, glass bottles, and jerrycans were obtained from the packaging distributors. Other materials consisting of chemicals used for analysis, such as KOH, iodine, O_2_, NaOH, and other chemicals, were obtained from chemical manufacturers. The specifications of the chemicals used for analysis are proanalysis grade. The equipment used consisted of equipment for the wet rendering extraction method, such as scales, measuring tools, steamers, stoves, and presses, as well as equipment for the dry rendering extraction method, such as scales, measuring tools, tanks/pans, stoves, stirrers, and filter tools.

### 2.2. Extraction Methods

There are two extraction methods used: wet and dry extraction. Wet rendering consisting of steaming, pressing, and filtering was adopted from Febrianto and Sudarno [8], whereas dry rendering consisting of melting (including agitation) and filtering was adopted from Ayu et al. [7], Amri et al. [24], and Fortuna Ayu et al. [25].

#### 2.2.1. Dry Rendering Method

For the dry rendering method, 4 kg of raw materials (belly or trimming from Pangasius fish) are cleaned and then heated in a stainless steel pan over low heat at a temperature of 75°C ± 5°C for 3.5 h until the oil liquid separates from the belly or trimming from Pangasius fish. Furthermore, coarse (200 mesh) and fine (400 mesh) filtration are carried out sequentially to produce crude Pangasius fish oil that is free from dirt and clear. The crude Pangasius fish oil is then packaged using 200 mL plastic bottles and stored in a clean and dry room with an ambient temperature of 27°C–30°C.

#### 2.2.2. Wet Rendering Method

Unlike the dry rendering method, in the wet rendering method, 4 kg of raw materials (belly or trimming from Pangasius fish) are cleaned and then steamed in a stainless steel pan over low heat at a temperature of around 65°C ± 5°C for 5 h, then pressed using a hydraulic press until liquid oil and solids (cake) are produced. The liquid oil is then left in a transparent container until two layers are formed, namely water and crude Pangasius fish oil. The crude Pangasius fish oil is then packaged using 200 mL plastic bottles and stored in a clean and dry room with an ambient temperature of 27°C–30°C.

The study examined two main treatment categories: extraction method (A), which included dry rendering (A1) and wet rendering (A2), and the types of byproducts from the Pangasius fillet industry (B), comprising trimming (B1) and belly (B2).

  A1B1: crude oil extraction from trimming using the dry rendering method.
  A1B2: crude oil extraction from the belly using the dry rendering method.
  A2B1: crude oil extraction from trimming using the wet rendering method.
  A2B2: crude oil extraction from the belly using the wet rendering method.

### 2.3. Characterization of Crude Pangasius Oil

The characterization of crude Pangasius oil consists of yield and analysis of the physicochemical properties of crude oil from industrial byproducts of Pangasius fillets, including free fatty acids (FFAs), acid value, PV, IV, and saponification value. The analysis was performed in duplicates. The final stage is further characterization for the best treatment, which includes physicochemical, microbiological, and metal property analyses using the Official Methods of Analysis of Association of Analytical Communities (AOAC) internasional method [26], and identification of value-added crude Pangasius oil extract using Hayami method.

#### 2.3.1. Yield of Crude Pangasius Oil Extraction

The percentage yield of crude Pangasius oil was analyzed using the equation of the ratio of the weight of crude Pangasius oil produced (g) to the weight of the sample belly or trimming from Pangasius fish used (g), then multiplied by 100% [27].

#### 2.3.2. FFAs of Crude Pangasius Oil

The FFAs of crude Pangasius oil were analyzed using the AOAC method [28] by extracting FFAs using a Soxhlet apparatus. The crude Pangasius oil obtained was then weighed for 20–40 mg of NaOH 0.5 N in methanol and heated in a water bath for 20 min. BF_3_ 20% (2 mL) was added after it was cooled. Two mL saturated NaCl and 1 mL hexane were also added and shaken until homogeneous. The hexane layer was transferred with a dropper pipette into a tube containing 0.1 g of anhydrous Na_2_SO_4_ and left for 15 min. The liquid phase was separated and injected into the GC. Identification of FFAs was performed by injecting methyl ester in gas chromatography with a mobile phase and pressurized nitrogen for 20 mL per minute. A capillary column, a Quadrex fused silica capillary column 007 cyanopropyl methyl silica, 60 m in length and 0.25 mm in diameter, was used.

#### 2.3.3. Acid Value of Crude Pangasius Oil

The acid value of crude Pangasius oil was analyzed using the AOAC method [26]. A total of 5–10 g of crude Pangasius oil is weighed and put into an Erlenmeyer flask, then 50 mL of 95% ethanol that has been neutralized is added. Heat until boiling (±10 min) while stirring, then cool to room temperature. Next, 3–5 drops of 1% phenolphthalein indicator are added and titrated with 0.1 N KOH solution until a pink color is formed that lasts for 30 s. The volume of KOH used is recorded.

#### 2.3.4. Peroxide Value of Crude Pangasius Oil

The PV of crude Pangasius oil was analyzed using the AOAC method [26]. A total of 5 g of crude Pangasius oil was put into a 250 mL Erlenmeyer flask, then added with 30 mL of acetic–chloroform acid solution (3:2) and shaken until dissolved. Saturated KI solution (0.5 mL) was added to the Erlenmeyer in sealed conditions and left for 1 min while shaken. After that, 30 mL of distilled water was added and titrated with sodium thiosulfate solution (Na_2_S_2_O_3_ 0.01 N) until the yellow color almost disappeared. A total of 0.5 mL of 1% starch solution was added and titrated again until the blue color began to disappear. The peroxide value was expressed in milliequivalents of peroxide in every 1000 g of the sample.

#### 2.3.5. Iodine Value of Crude Pangasius Oil

The IV of crude Pangasius oil analysis was conducted based on the SNI ISO 3961:2015 method [29]. Around 0.1 g of crude Pangasius oil was weighed into 250 mL of Erlenmeyer, then 10 mL of chloroform and 25 mL of iodine–bromide reagent were added and left in a dark room for 30 min. A 15% KI solution and distilled water were added for 10 mL and 50–100 mL, respectively, then immediately added with sodium thiosulfate solution (Na_2_S_2_O_3_ 0.1 N) until the color turned into pale yellow. A 2 mL of starch solution was added, and the titration continued until the blue color disappeared. A blank solution was made from 25 mL of iodine–bromide reagent, and 10 mL of 15% KI was diluted with 100 mL of distilled water, which had been boiled and titrated with sodium thiosulfate solution. The amount of sodium thiosulfate used for titrating blanks minus the titration in the sample was equivalent to the amount of iodine bound by fat or oil.

#### 2.3.6. Saponification Value of Crude Pangasius Oil

The saponification value of crude Pangasius oil was analyzed using the AOAC method [26]. A total of 2–5 g of crude Pangasius oil was weighed and put into an Erlenmeyer flask, then 50 mL of 0.5 N alcoholic KOH solution was added with a volumetric pipette. A blank with the same procedure without an oil sample was also prepared. Then the sample was connected to the reverse condenser and heated on a hot plate, then refluxed for 30 min until complete saponification (clear solution). The reflux result was then cooled to room temperature. The reverse condenser was removed, and 3–5 drops of phenolphthalein indicator were added, then the excess KOH was titrated with 0.5 N HCl until the pink color disappeared. The volume of HCl used for the sample and blank was recorded.

#### 2.3.7. Fatty Acid

The fatty acid of crude Pangasius oil was analyzed using ISO 12966‐2-2011 [28]. Fat or oil is hydrolyzed using a base (NaOH in methanol), followed by methylation into fatty acid methyl esters using sodium methylate in methanol. The fatty acid content is determined by comparing the outer area of the standard with the area of each fatty acid component present in the sample.

#### 2.3.8. Microbiological Test

*Salmonella* sp. were analyzed using the testing method described in Indonesia National Standard (SNI) 01-2332.2:2006 [30]. The testing method for *Salmonella* sp. includes pre-enrichment, enrichment, and cultivation on designated media. The underlying principle of the analysis involves the growth of bacteria in liquid media, specifically Triple Sugar Iron Agar. The quantification of colonies is determined by the count of positive tubes following incubation at a defined temperature and duration.

*Escherichia coli* were analyzed using the testing method described in SNI 2332.1:2015 [31]. The test was conducted using the conventional method (most probable number [MPN]-based) analysis principle, which consists of the following stages: initial estimation with gas/acid from lactose fermentation, followed by culture confirmation under selective conditions to separate coliforms and *E. coli*. The colony count was determined using the MPN method.

#### 2.3.9. Heavy Metals Test

Mercury (Hg), cadmium (Cd), arsenic (As), tin (Sn), and lead (Pb) were analyzed using the testing method described in SNI 2354.23:2021 [31]. Analysis is conducted concurrently utilizing inductively coupled plasma mass spectrometry (ICP-MS) technology. This method allows for the identification and quantification of multiple metal elements with remarkable precision, outstanding sensitivity (detectable down to ppt–ppb), and optimal analytical efficiency, as it can assess several metals at once. The testing protocol encompasses sample preparation, standardization, quantification, and validation. Sample preparation, involving acid digestion or ashing to transform the solid sample into a liquid solution. Standardization is performed to prepare standard calibration solutions for every target metal. The analysis using ICP-MS involves the introduction of the sample solution by spraying it into the plasma, where ions are segregated according to their mass-to-charge ratio. Quantification is carried out by comparing the sample signal with the standard and matrix correction and internal standard. Validation was performed to determine the parameters: linearity, accuracy, precision, detection limit, and quantification.

#### 2.3.10. Color (Lovibond Yellow and Red 5.25″ Cell) Analysis

The color of crude Pangasius oil was analyzed using the Lovibond Tintometer method according to AOCS Official Method Cc 13b-45 [32]. Samples were carefully filtered to remove impurities and transferred into a 5.25-inch optical glass cell. The color was measured by visually matching the oil sample against calibrated Lovibond Red and Yellow glass standards, and the results were expressed as Lovibond Yellow and Red units (5.25″ cell). The intensity of each primary color required to match the sample's color is recorded. The result is expressed as a combination of Lovibond Red and Yellow units.

#### 2.3.11. Melting Point Test

The melting point is determined by the Fish Oil Melting Point Test Method, according to ISO 6321:2021 [33]. The principle employed involves identifying the temperature at which a column of solidified oil starts to “slip” upward in a capillary due to the melting process. The equipment utilized includes a glass capillary tube, a water bath with temperature regulation, a digital or mercury thermometer, and ice. The testing procedures are as follows: Heat the fish oil until it reaches a liquid state, then transfer it into a small capillary tube (approximately 1 mm in diameter). Cool the capillary (for instance, in a refrigerator set at 0°C–5°C) until the oil solidifies. Next, attach the capillary to the thermometer and immerse it in the water bath. Gradually increase the temperature of the water bath at a rate of approximately 1°C per minute. Record the temperature at which the fat or oil column begins to slip upwards as the melting point.

#### 2.3.12. Anisidine Value (AnV) Determination

The p-AnV of crude Pangasius oil was measured according to ISO 6885:2016 [34] using a spectrophotometric technique. A specified quantity of oil was dissolved in iso-octane to create a test solution. The absorbance at 350 nm was measured both prior to and following the reaction with a p-anisidine reagent solution in glacial acetic acid. The increase in absorbance is indicative of the concentration of aldehydic secondary oxidation products, primarily consisting of 2-alkenals and 2,4-dienals. The AnV was calculated using the formula:

(1) $$\begin{array}{c} \text{Anisidine Value}=25\times \frac{\left(1.2\times \text{As}-\text{Ab}\right)}{m}, \end{array}$$

where As = absorbance of the reacted solution at 350 nm, Ab = absorbance of the blank solution at 350 nm, and *m* = mass of the test sample (g).

#### 2.3.13. Vitamin Content Test

Vitamin A content analysis was conducted according to AOAC [35]. Initially, the sample was treated with ethanolic potassium hydroxide for saponification to facilitate the hydrolysis of retinyl esters. Then, extraction was conducted using an organic solvent, hexane, to effectively isolate retinol. The extract obtained was concentrated and analyzed with high-performance liquid chromatography (HPLC), using a reversed-phase C18 column and an ultraviolet (UV) detector operating at 325 nm. The calculation of retinol was accomplished through the application of external calibration utilizing standards of retinol.

Vitamin D content analysis was conducted according to Standards EN 12821:2009 [36], using HPLC. The initial phase involved the saponification of the sample using ethanolic potassium hydroxide, intended to release free vitamin D, followed by a hexane extraction process to isolate unsaponifiable matter. The obtained extract was purified with solid-phase extraction (SPE) prior to analysis through HPLC, which was equipped with a UV detector set at a wavelength of 265 nm.

Vitamin K content analysis was conducted in accordance with the AOAC Official Method 999.15 [37], with particular modifications adapted for the complex matrices associated with fish oil. An estimated amount of 100–200 mg of the oil sample was carefully measured, combined with an internal standard (K_1_-d_7_), and subjected to saponification using ethanolic KOH that included ascorbic acid, all performed in a nitrogen environment at a temperature of 50°C–60°C for a period of 30 min. The unsaponifiable fractions were subsequently isolated through hexane extraction, neutralized, dried, and subsequently subjected to silica SPE to facilitate the removal of triglycerides. The resulting eluate was subsequently evaporated, reconstituted in the mobile phase, and analyzed using HPLC. This process involved postcolumn zinc reduction and fluorescence detection, with an excitation wavelength set at 244 nm and an emission wavelength at 430 nm. Calibration procedures were performed utilizing standards of phylloquinone and menaquinone, with the concentrations expressed as μg/g of oil. Moreover, the findings were transformed into international units (IU/g) for K_1_, utilizing the conversion factor of 1 IU = 0.33 μg of phylloquinone.

### 2.4. Statistical Analysis

Statistical analysis of the characterization results was performed using analysis of variance (ANOVA) with a two-factorial completely randomized design [38], where the first factor was the extraction method (wet and dry rendering methods) and the second factor was the type of byproduct from the Pangasius fillet industry (trimming and belly). Analysis of variance was performed using IBM SPSS Statistics Version 26 for Windows. The test results showed that the calculated F was greater than or equal to the F table; therefore, further tests were carried out using Duncan's multiple range test (DMRT) at a 5% level to determine the differences in the effect of each treatment.

### 2.5. Business Potentials Based on Value-Added

The identification of value-added crude Pangasius oil extract using an analytical tool, namely the Hayami method (Table 1). The Hayami method is generally carried out in three stages, namely the stage of calculating output, input, and prices; the stage of calculating income and profits; and the final stage of calculating the percentage of value-added [39].

## 3. Results and Discussion

### 3.1. The Visual and Physical Properties of Crude Pangasius Oil

**Figure 1:**
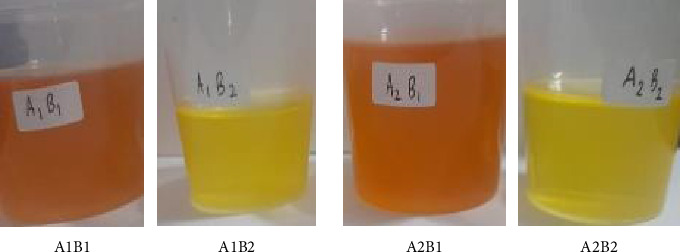
The color of crude Pangasius oil when heated at temperatures above 29°C.

The results of visual identification of crude oil obtained from the byproducts of the Pangasius fillet industry were clear yellow and orange (Figure 1) with a solid texture and distinctive aroma (Pangasius fish aroma). Figure 1 shows that the type of raw material influences the color of crude Pangasius oil when heated to temperatures above 29°C. Crude Pangasius oil made from trimmings was orange (A1B1 and A2B1), whereas crude Pangasius oil made from the belly was yellow (A1B2 and A2B2). The raw material for trimming is leftover meat, which is orange, whereas the belly is yellow stomach fat. Extraction methods, both wet rendering and dry rendering, can affect the color of catfish oil. The dry method with high temperature causes a more intensive Maillard reaction, resulting in a darker oil color, while the wet rendering method with lower temperature produces a brighter oil color because it minimizes the browning reaction [40, 41]. The dry method can cause degradation of natural pigments and affect the intensity of the oil color, while the wet rendering method helps reduce the transfer of pigments from tissue to oil due to the presence of a water phase as a barrier [40, 41]. Carotenoids and other pigments are more easily oxidized in the dry method, resulting in changes in oil color [16, 42], while the wet rendering method can maintain pigment stability better [43], resulting in a more consistent oil color.

### 3.2. The Chemical Properties of Crude Pangasius Oil

The chemical characterization of crude Pangasius oil consisted of FFAs, acid value, PV, IV, and saponification value. Extraction methods, both wet rendering and dry rendering, can affect the chemical characteristics of Pangasius oil; this is shown in several aspects, namely the effect of temperature and process time, interaction with other components, and material characteristics [40, 41].  Table 2 shows the chemical properties of crude Pangasius oil from the industrial byproducts of Pangasius fillets.

Based on the chemical characteristics in Table 2, the quality of crude Pangasius oil extracted from fillet industry byproducts differs significantly depending on the extraction method (dry or wet rendering) and the raw material source (trimming or belly). In order to facilitate a more pertinent assessment, the discourse references SNI 7950:2013 concerning crude sardine oil and the standards set forth by IFOMA (International Fishmeal and Fish Oil Manufacturers Association) regarding the quality of crude fish oil.

#### 3.2.1. FFAs

FFAs are fatty acids that are in the free acid group and are not bound to triglycerides and are characterized by their potential to produce rancid properties in oil [45]. The identification results in Table 2 show that the FFA content of crude Pangasius oil ranged from 0.0550 ± 0.0071% (A2B2 treatment) to 0.880 ± 0.9970% (A1B2 treatment). All samples analyzed demonstrate FFA concentrations significantly below the maximum threshold of 3% as outlined by both SNI 7950:2013 and IFOMA for crude fish oil. The sample A2B2 exhibited the lowest FFA level at 0.055%, suggesting minimal hydrolytic rancidity, which can be attributed to efficient wet rendering processes and the superior quality of the belly tissue. This suggests A2B2 oil has high freshness and stability. Lower FFA levels indicate better physical characteristics of the oil, such as aroma and taste [25].

This means that crude oil extraction from the belly using the wet rendering method shows better FFA levels than the other treatments. Differences in extraction methods and materials certainly influenced the levels of FFAs in the crude Pangasius oil produced [7, 46, 47]. The dry method causes protein denaturation and facilitates oil release but can increase the formation of FFAs [45], while the wet rendering method with the addition of water helps separate the oil from the tissue matrix and reduces thermal damage [40]. FFA values tend to be higher in the dry method due to more intensive triglyceride hydrolysis [45]. The analysis of variance showed that the F-count value (1.373) was greater than the F-table value (0.05), with a significance value of 0.372, indicating that there was a real difference in the FFA values for each treatment. Analysis of variance was continued with the Duncan test, and a significant value of 0.135 was obtained for all treatments in the same column, indicating that there was no real difference in the FFA values for each treatment.

#### 3.2.2. Acid Value

Acid value was used to measure the acidity of a particular chemical substance. This is the amount of base (usually potassium hydroxide [KOH]) expressed in mg of KOH required to neutralize the acidic elements in 1 g of the sample [48]. As shown in Table 2, the acid value of crude Pangasius oil ranged from 0.6150 ± 0.2616 mL NaOH per 100 g oil (A1B2 treatment) to 1.7450 ± 0.0212 mL NaOH per 100 g oil (A1B1 treatment). This means that crude oil extraction from the belly using the dry rendering method shows a better acid value than the other treatments. Though typically more relevant to refined oils, the acid values observed were all within acceptable ranges (< 3 mL NaOH/100 g oil) based on IFOMA guidelines. The minimum acid value was observed in A1B2 (0.615 mL NaOH/100 g oil), which further corroborates its exceptional oil quality in comparison to other dry rendering treatments.

Similar to the FFA levels in crude Pangasius oil, the differences in extraction methods and extracted materials certainly influence the acid value of the crude Pangasius oil produced [7, 46, 47]. Lower acid values indicate better physical characteristics of the oil, such as aroma and taste [25]. The dry method with high temperature causes increased triglyceride hydrolysis, which produces higher FFAs, thus increasing the acid value, while the wet rendering method with lower temperature can minimize the formation of FFAs, thus producing a lower acid value [40, 41]. Longer processing time in the dry method increases hydrolysis reactions that affect the acid value, and the basic method using water can help reduce direct contact with heat, minimizing changes in acid value [39, 41]. The analysis of variance showed that the F-count value (14.139) was greater than the F-table value (0.05), with a significance value of 0.014, indicating that there was a real difference in the acid values for each treatment. The analysis of variance was continued with the Duncan Test, and it was found that the A1B1 treatment was significantly different from the other treatments because they were in different columns. A1B2, A2B1, and A2B2 treatments were not significantly different because they were in the same column.

#### 3.2.3. The PV

The PV was used to determine the level of damage to the structure of crude Pangasius oil [13]. The identification results As shown in Table 2, the PV of crude Pangasius oil ranged from 5.8500 ± 0.3111 mEq O_2_ per 100 g of oil (A1B2 treatment) to 20.4000 ± 0.1414 mEq O_2_ per 100 g of oil (A1B1 treatment). This means that crude oil extraction from the belly using the dry rendering method shows a better PV than the other treatments. A high PV indicates damage to crude Pangasius oil, which is characterized by the appearance of rancidity formed by aldehydes. Hydroperoxides can break unsaturated bonds into saturated bonds. The formation of hydroperoxides is influenced by several factors, such as the heating treatment and freshness of the ingredients [7, 46, 47].

As per SNI 7950:2013, the maximum permissible PV for crude sardine oil is 5 mEq O_2_/kg. Only A1B2 (5.85 mEq/kg) slightly exceeds this limit, while A2B2 (11.25 mEq/kg) and A2B1 (11.955 mEq/kg) are clearly above the standard. A1B1 (20.40 mEq/kg) is significantly higher and may indicate early oxidative degradation. This suggests that oil from trimming (especially via dry rendering) may be more prone to oxidation unless handled or processed quickly.

The PV increases as the heating temperature increases during extraction and decreases as the temperature decreases to 95°C [49]. A low PV can cause the formation rate of new peroxides to be lower than the rate of degradation of the peroxides into other compounds. Peroxides degrade quickly and react with other substances [48]. The dry method, which uses high temperatures for a longer time, can increase fat oxidation and produce higher PVs, while the wet rendering method, which uses lower temperatures for a shorter time, can minimize oxidative damage to the oil [40, 41, 45]. The analysis of variance showed that the F-count value (31.322) was greater than the F-table value (0.05), with a significance value of 0.003, indicating that there was a real difference in the PVs for each treatment. The analysis of variance was continued with the Duncan Test and showed that the A1B1 treatment was significantly different from the A1B2 treatment and significantly different from the treatments of A2B1 and A2B2 because it was in a different column. The A2B1 and A2B2 treatments were not significantly different because they were in the same column.

The PV serves as a crucial measure of primary lipid oxidation, indicating how many unsaturated fatty acids (UFAs) have been subjected to peroxidation. When examining crude Pangasius oil derived from byproducts, the elevated PV found in A1B1 (20.400 ± 0.1414 mEq O_2_/kg) in comparison to A1B2 (5.850 ± 0.3111 mEq O_2_/kg) underscores notable oxidative variations between trimming oil and belly oil produced under dry rendering conditions. One of the factors contributing to variations in PVs is the unsaturation of fatty acids and their oxidative stability. Fats that are trimmed typically have a higher concentration of PUFAs, which include omega-3 fatty acids (EPA, DHA) and omega-6 fatty acids. These PUFAs are particularly susceptible to oxidation, especially during thermal processing in the absence of moisture, as seen in dry rendering. The double bonds present in PUFAs exhibit high reactivity to oxygen, resulting in the production of hydroperoxides, which can be identified in PV tests. Although belly fat is also abundant in unsaturated fats, it tends to contain a greater proportion of monounsaturated fats, such as oleic acid, and experiences somewhat less surface oxidation due to its larger lipid structure and denser tissue arrangement. The markedly higher PV in the oil from trimmings (A1B1) during dry rendering is likely attributed to a greater presence of PUFAs and increased surface exposure while heating. PUFAs are particularly susceptible to oxidation because of their multiple double bonds, especially when moisture and antioxidants are lacking during thermal processing [50].

#### 3.2.4. The IV

The IV indicates the degree of unsaturation of the fatty acid components of crude Pangasius oil. Oils with a high unsaturated fatty acid content bind more significant amounts of iodine and form saturated compounds [38]. As shown in Table 2, the IV of crude Pangasius fish oil ranged from 60.8000 ± 1.2728 g I_2_ per 100 g of oil (A1B1 treatment) to 88.3000 ± 3.2527 g I_2_ per 100 g of oil (A2B1 treatment). Although SNI 7950:2013 does not specify a maximum IV, the IV is an important measure of unsaturation. All tested samples were below 140 g I_2_/100 g oil, a standard reference for fish oil. A2B2, with an IV of 86.50 g I_2_/100 g, exhibited the highest value, suggesting a greater content of PUFAs, which is nutritionally advantageous but may necessitate antioxidant stabilization during storage.

Iodine numbers can differ due to the effect of temperature on double bonds of fatty acids. Dry method with high temperature can cause damage to double bonds in UFAs, reducing iodine number, while the base method with lower temperature is better in maintaining double bonds so that iodine number is more stable [40, 41, 45]. Dry method is more susceptible to oxidation which can break double bonds and reduce iodine number, while the base method with water as a barrier can reduce contact with oxygen so that it protects double bonds more [40, 41, 45]. Longer process time in dry method increases the possibility of oxidation and isomerization which affects iodine number, while the base method with shorter process time can minimize changes in the structure of UFAs [40, 41, 45]. The analysis of variance showed that the F-count value (83.801) was greater than the F-table value (0.05), indicating that there was a real difference in the IVs for each treatment. The analysis of variance continued with the Duncan Test and showed that the treatments of A1B1 and A1B2 were significantly different from those of A2B1 and A2B2 because they were in a different column. The A2B1 and A2B2 treatments were not significantly different because they were in the same column. This is the same as the A2B1 and A2B2 treatments, which were not significantly different because they were in the same column.

#### 3.2.5. The Saponification Value

The saponification value indicates the size of the fatty acid molecules contained in crude Pangasius oil, where oils composed of short-carbon chain fatty acids have a relatively small molecular weight, so they have a large amount of saponification, and vice versa [7]. As shown in Table 2, the saponification value of crude Pangasius oil ranged from 211.8350 ± 0.8132 mg KOH per gram of oil (A1B2 treatment) to 259.3050 ± 42.9567 mg KOH per gram of oil (A2B1 treatment). This means that crude oil extraction from the belly using the dry rendering method shows a better saponification value than the other treatments. The higher the saponification value, the lighter the average molecular weight of the triglycerides and the lower the average length of the fatty acids, and vice versa. The saponification values, while not directly regulated by SNI 7950:2013, offer valuable information regarding the average molecular weight of the fatty acids present in the oil. A2B1, with a value of 259.305 mg KOH/g, was the highest, indicating a predominance of shorter-chain fatty acids. A1B2, at 211.835 mg KOH/g, is within the typical range (180–210 mg KOH/g) for fish oil, suggesting its appropriateness for further refinement or industrial applications.

The analysis of variance showed that the F-count value (2.602) was greater than the F-table value (0.05), with a significance value of 0.189, indicating that there is a significant difference in the saponification values for each treatment. The analysis of variance was continued with Duncan's test, and a significance value of 0.101 was obtained. All treatments were in the same column, indicating that there was no significant difference in saponification values for each treatment.

Overall, A1B2 (crude oil obtained from the belly through the dry rendering technique) exhibits the most advantageous combination of characteristics: moderate FFA and acid values, acceptable peroxide levels close to the SNI threshold, along with favorable iodine and saponification values. Although there is a minor exceedance of PV, the overall profile indicates that it is the most feasible option for commercial development, especially if further refining processes (such as degumming or antioxidant treatment) are implemented.

Refining of crude Pangasius oil from byproducts of the Pangasius fillet industry needs to be performed to identify the characteristics of refined Pangasius oil and compare them with standards set by the country or the world. The refining process mainly comprises degumming, deacidification, decoloration, and deodorization [51]. However, the identification results in this study showed that the dry rendering method was the best extraction method for crude Pangasius oil from byproducts of the Pangasius fillet industry (trimming and belly). The mechanism by which treatment affects FFA levels during the extraction of crude Pangasius oil from byproducts involves several key factors related to extraction methods, temperature, and time. Dry rendering generally results in lower FFA levels than wet rendering, whereas solvent extraction can be optimized to balance the yield and oil quality. Careful control of these parameters is crucial for producing high-quality Pangasius oil with minimal FFAs. Different extraction methods, temperatures, and times significantly influence the FFA content in the extracted oil [52, 53].

The dry rendering method involves heating fish byproducts without water. This typically results in lower FFA levels compared to wet methods, because the absence of water minimizes hydrolysis reactions that can lead to the formation of FFAs. Wet rendering with water added to the extraction process can increase the extraction yield, which often leads to higher FFA levels due to the hydrolysis of triglycerides into FFAs. The presence of water facilitates enzymatic reactions that can degrade the oil quality, resulting in a higher FFA content.

Extraction temperature plays a crucial role in determining the quality of the extracted oil. Higher temperatures can increase the extraction rate but may also accelerate the degradation of triglycerides into FFAs. The duration of the extraction process also affected FFA levels. Prolonged extraction times can lead to increased FFA formation owing to the extended exposure of triglycerides to hydrolytic conditions. Therefore, optimizing the extraction time is essential to minimize FFA content while maximizing oil yield.

To evaluate the quality parameters of the Pangasius oil produced, benchmarks were carried out with several standards and literature as described as follows (Table 3).

### 3.3. The Physical, Chemical, Microbiological, and Metal Properties of Crude Pangasius Oil From the Best Treatment

The best extraction treatment was oil from the belly using the dry rendering method (A1B2). In the best treatment, further characterization of the physical and chemical properties of crude Pangasius oil will be carried out.  Table 4 shows the physical, chemical, microbiological, and metal properties of crude Pangasius oil.

The characterization of crude Pangasius oil from the best-performing treatment (A1B2: dry rendering of belly byproducts) was evaluated against relevant standards for crude fish oil, notably SNI 7950:2013 (crude sardine fish oil), IFOMA (International Fishmeal and Oil Manufacturers Association), and Codex Alimentarius guidelines.

#### 3.3.1. Physical Properties

**Figure 2:**
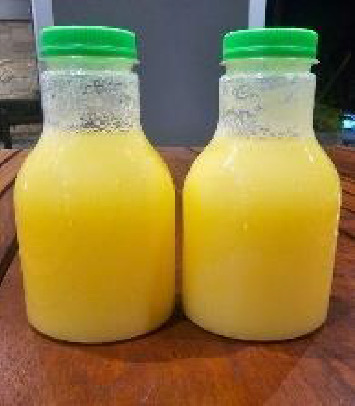
Crude Pangasius oil from the belly is solid at room temperature (between 25°C and 29°C).

The Lovibond color values (Yellow: 65.00; Red: 2.90) are typical for crude fish oils and reflect natural pigment compounds such as carotenoids. This shows that the crude Pangasius oil made from the belly is clearly yellow, as shown in Figures 1 and 2. While SNI 7950:2013 does not prescribe specific color thresholds, similar values have been reported in crude marine oils [57].

The melting point of 33°C is within the range reported for semi-solid tropical fish oils, making it suitable for functional or processed food formulations. The melting point of crude Pangasius oil is 31–42°C. This means that the melting point of crude Pangasius oil from the results of this study is within the melting point range of crude Pangasius oil from Widowati's research [59]. The high melting point of crude Pangasius oil is indicated by the high content of UFAs contained in crude Pangasius oil, namely myristic acid (7.74%), palmitic acid (40.70%), stearic acid (9.90%), oleic acid (21.20%), linoleic acid (10.60%), and linolenic acid (6.83%) (Table 5). This means that the higher the unsaturated bond, the lower the melting point [59].

#### 3.3.2. Fatty Acid Profile

The fatty acid composition of the oil is primarily characterized by the following: omega-3 at 1007.30 mg/100 g, omega-6 at 12,329.60 mg/100 g, omega-9 at 36,268.55 mg/100 g, DHA at 173.60 mg/100 g, and EPA at 176.00 mg/100 g. While the omega-3 content is moderate compared to marine oils like sardine or cod liver oil, it still offers nutritional benefits. Crude sardine oil, as referenced in SNI 7950:2013, typically has higher levels of EPA and DHA. However, Pangasius oil can serve as a viable alternative source of omega-3s [57, 59]. The high omega-9 content, primarily from oleic acid at 21.20%, also contributes to enhanced oxidative stability and overall nutritional value.

The oil's fatty acid composition is its most significant competitive advantage, resulting in an impressive nutritional profile. It contains a notably high level of omega-9 fatty acids at 36,268.55 mg per 100 g, with oleic acid making up 21.20% of this profile, a characteristic comparable to heart-healthy olive oil [60]. Additionally, it serves as a substantial source of omega-6 (12,329.60 mg per 100 g) and omega-3 (1007.30 mg per 100 g) fatty acids, including essential ones like linoleic and linolenic acid. The oil's value is further enhanced by its content of beneficial long-chain omega-3s, providing 173.60 mg per 100 g of DHA and 176.00 mg per 100 g of EPA. These compounds are renowned for their critical roles in supporting brain health and cardiovascular function and reducing inflammation [60, 61].

The AnV is recorded as 5.88. This value measures secondary oxidation products and is a key indicator of oil stability and freshness. An AnV of 5.88 is an excellent result for crude fish oil. The Global Organization for EPA and DHA omega-3s [55] sets a maximum limit of 20 for finished fish oils, indicating this crude oil has low levels of secondary oxidation [57, 62]. Its unique fatty acid profile, especially the high concentration of oleic acid (omega-9) combined with significant amounts of omega-3s (including EPA and DHA) and omega-6s, makes it a versatile nutritional ingredient.

The results of microbiological and heavy metal identification showed values that followed Indonesian national food standards. The analysis shows that harmful bacteria such as *Salmonella* sp. were negative in a 25 g sample, and *E. coli* levels were less than 3 MPN/g. These results indicate high hygienic quality during processing. The absence of detectable heavy metals and harmful microbes, coupled with a low oxidation value for a crude product, makes it a safe and reliable raw material [56]. But crucially, toxic heavy metals, including mercury (Hg), cadmium (Cd), arsenic (As), tin (Sn), and lead (Pb), were “not detected.” The table provides the specific, low limits of detection (e.g., 0.0008 mg/kg for mercury), confirming the tests were highly sensitive. Overall, given its robust profile, this oil is an excellent candidate for use in dietary supplements, functional foods, animal feed enrichment, and potentially the cosmetics industry. As a “crude” oil, it shows exceptional quality, suggesting that further refining could yield a premium product with even broader applications.

#### 3.3.3. Oxidation Level

The AnV of 5.88 falls within acceptable limits for crude fish oil (< 10), indicating minimal secondary oxidation [55]. Though SNI does not specify AnV, it is commonly used as a marker for aldehyde formation postprimary oxidation and is widely accepted by Codex and the oil industry.

#### 3.3.4. Chemical Properties

The predominance of palmitic acid (40.70%), oleic acid (21.20%), linoleic acid (10.60%), and stearic acid (9.90%) aligns with profiles typical of fish oils. Minor short-chain acids such as butyric (C4:0) and capric (C10:0) were not detected or present in negligible amounts, suggesting low rancidity and favorable sensory characteristics.

The fatty acid profile of the best crude Pangasius oil sample (A1B2) revealed a predominance of palmitic acid (C16:0) at 40.70%, oleic acid (C18:1) at 21.20%, and linoleic acid (C18:2) at 10.60%. These findings are consistent with previous studies that characterized several marine fish oils as possessing a mixed fatty acid profile, including both saturated fatty acids (SFAs) and UFAs, especially monounsaturated fatty acids (MUFAs) and PUFA [56].

Palmitic acid, although classified as a SFA, plays a significant role in enhancing oil stability due to its ability to resist oxidative degradation, which is advantageous for both shelflife and industrial processing. Nevertheless, its elevated levels require a careful balance with UFAs to ensure nutritional adequacy, particularly in the context of edible oil applications.

The fatty acid composition observed also sheds light on the oxidative characteristics of the oil. The belly section, which exhibited increased amounts of UFAs, demonstrates a greater vulnerability to primary oxidation, as evidenced by its elevated PV. This underscores the necessity for protective storage measures, antioxidant supplementation, or postextraction refining, particularly if the oil is intended for human consumption.

#### 3.3.5. Vitamin Content

The crude oil derived from Pangasius, specifically obtained through the optimal method of dry rendering of belly byproducts, was analyzed and found to contain vitamin A (161.65 IU/g), vitamin D (192.40 IU/g), and vitamin K (3.20 IU/g). Although these values are lower than those found in pharmaceutical-grade oils like cod liver oil, they remain nutritionally significant, particularly for applications in functional foods. The vitamin A concentration in Pangasius oil is less than that of cod liver oil, which generally ranges from 850 to 2000 IU/g [59]. In terms of vitamin D, the Pangasius oil contains 192.40 IU/g, which is lower than the levels found in cod liver oil (400–1000 IU/g), yet it remains a valuable source. Regarding vitamin K, while fish oil is not generally recognized as a primary source, the measured level of 3.20 IU/g (approximately 3 μg/g) is higher than the 0.896 IU/g of vitamin K produced from sprat (*Sprattus sprattus*) [62]. While SNI 7950:2013 does not mandate vitamin thresholds, their presence enhances the oil's nutraceutical value. Although crude Pangasius oil does not reach the vitamin concentrations of specialized fish oils, it offers a nutritionally relevant profile that, when refined and stabilized, could facilitate its use in fortified foods or dietary oil blends.

#### 3.3.6. Microbiological Quality

The results of microbiological identification showed values that followed Indonesian national food standards. No *Salmonella* sp. was detected, and *E. coli* was found at < 3 MPN/g, fulfilling both SNI 7388:2009 and Codex microbiological standards for fish oil safety [54]. These results indicate effective hygienic rendering and handling practices.

#### 3.3.7. Heavy Metal Contamination

The results of heavy metal identification showed values that followed Indonesian national food standards. All tested heavy metals, Hg, Cd, As, Sn, and Pb, were not detected, remaining below method detection limits (e.g., Pb < 0.0004 mg/kg). These levels are significantly lower than Codex maximum limits for edible oils (Pb: 0.1–0.3 mg/kg, Hg: 0.5 mg/kg, and Cd: 0.05 mg/kg) [54]. Thus, the crude Pangasius oil is safe with respect to toxic metal content and environmentally compliant.

### 3.4. The Yield of Extraction of Crude Pangasius Oil

Extraction yield is one of the factors that has a very important influence on industrial feasibility. The yield of extraction is shown in Table 5.

The highest yield was obtained with the A1B2 treatment (crude oil extraction from the belly using the dry rendering method) at 25.02%. Whereas the A1B1, A2B1, and A2B2 treatments were 22.71%, 14.09% and 15.16%. The yield of dry rendering method was higher compared to the wet rendering method. The yield of dry rendering method were 25.02% and 22.71%, while the yield of wet rendering method were 15.16% and 14.09%. The yield of crude oil extraction from the belly was higher compared to crude oil extraction from the trimming.

### 3.5. Business Potentials Based on Value-Added

Value added is a change in value that occurs owing to changes in input in a production process [63]. Technical factors that affect value-added are production capacity, number of raw materials used, and labor. At the same time, the market factors that affect output are the price of output, work wages, price of raw materials, and value of other inputs. The concept of value-added is highly dependent on existing demand and often changes based on the value of a product that consumers want. The value-added analysis of crude Pangasius oil products is per year of production; the standard price used for raw materials, labor, and other input donations is the price at the processing or producer level. The average value of the added value from the production of crude Pangasius oil is presented in Table 6.

Table 6 shows that the conversion value for the crude Pangasius oil product was 0.25. The product conversion factor indicates that every 1 kg of fresh industrial byproduct of Pangasius fillets produces a crude Pangasius oil output of 0.25 L. The conversion factor value affects the output value of the product (U.S.$/kg). The average primary raw material (fresh industrial byproducts of Pangasius fillets) needed in one production process is 6600.00 kg per year. It will produce 1650.00 kg of crude Pangasius oil products per year. The value-added activity requires input donations from other materials (supporting materials), costing U.S.$ 0.18 for each kilogram of input (primary raw material) used. The supporting ingredients include packaging, electricity, water, gases, and utilities. The output value obtained using crude Pangasius oil products was U.S.$ 2.24/L. The value-added obtained was U.S.$ 1.62 with a ratio of 72.48% of the total output value. The income received by labor per kilogram of output amounted to U.S.$ 0.001338, or 0.0825% of the total value-added. The profit value is obtained from value-added minus direct labor income, so that the profit received for each kilogram of output is U.S.$ 1.62, or 72.42% of the total output value. The margin value is the difference between the output and input prices. For each cycle, the crude Pangasius oil production cycle had a margin of U.S.$ 1.80/L. A margin value of 90.08% is profit. In comparison, the remaining is divided into 0.0744% of the direct labor income margin, and 9.85% is the contribution of other inputs (supporting material costs) incurred from crude Pangasius oil production. This indicates that the greater the output produced, the greater the value-added obtained, and the more efficient the producer in trying, the greater the workforce's competitiveness [64].

## 4. Conclusion

This research successfully produced crude oil from Pangasius fillet byproducts, with dry extraction yielding a higher amount (25%) than wet extraction (15%). The resulting crude oil from dry extraction shows potential as a commercial food product due to its physicochemical characteristics and compliance with food standards with their characteristics of a melting point 33.00°C, vitamin A 161.65 IU/g, vitamin D 192.40 IU/g, and vitamin K 3.20 IU/g, and the three highest fatty acid compounds were palmitic acid (40.70%), oleic acid (21.20%), and linoleic acid (10.60%), and the value-added obtained is U.S.$ 1.62 per L of crude Pangasius oil.

The best characteristics of crude oil (A1B2) were an FFA content of 0.880%, an acid value of 0.615 mL NaOH/100 g oil, a PV of 5.85 mEq per 100 g oil, an IV of 65.53 g I_2_ per 100 g oil, and a saponification value of 211.835 mg KOH/g oil. This study could be a valuable contribution to the field of sustainable aquaculture processing. Further processing, such as refining, bleaching, and deodorizing, is recommended to improve the oil's purity, color, and odor, making it more suitable for food applications. Additionally, consider encapsulation techniques to protect the oil's nutritional content and improve its stability in food products.

## Figures and Tables

**Table 1:** Stages of calculation of value-added Hayami method (modified).

No.	Variable	Value
*I*	*Output, input, and price*	
1	Output (kg)	*A*
2	Raw material input (kg)	*B*
3	Labor input (DWH)	*C*
4	Conversion factors (input/output)	*D* = *B*/*A*
5	Field labor coefficient (DWH/kg)	*E* = *C*/*B*
6	Output price (U.S.$/kg)	*F*
7	Average labor wage (U.S.$/DWH)	*G*

*II*	*Profit and revenue (IDR/kg raw material)*	
8	Input price (U.S.$/kg)	*H*
9	Transaction fees (U.S.$/kg)	*I*
10	Output value (U.S.$/kg)	*J* = *D* ∗ *F*
11	Value-added (U.S.$/kg) value-added ratio (%)	*K* = *J* − *H* − *I* *L* = (*K*/*J*) ∗ 100%
12	Labor income (U.S.$/kg) Labor incentives (%)	*M* = *E* ∗ *G* *N* = (*M*/*K*) ∗ 100%
13	Profit (U.S.$/kg) Profit rate (%)	*O* = *K* − *M* *P* = (*O*/*J*) ∗ 100%

*III*	*Production factor services (U.S.$/kg of raw materials)*	
14	Margin (U.S.$/kg) Direct labor income (%) Other input donations (%) Company profits (%)	*Q* = *J* − *H* *R* = (*M*/*Q*) ∗ 100% *S* = (*I/Q*) ∗ 100% *T* = (*O*/*Q*) ∗ 100%

*Note:* Source: Hayami et al. [39]; U.S.$ = American dollar (value of 1 U.S.$ exchange rate IDR 15,955.30).

Abbreviation: DHW = daily human works.

**Table 2:** The chemical properties of crude Pangasius oil from Pangasius fillets industrial byproducts.

Treatments	Free fatty acids (%)	Acid value (mL NaOH/100 g oil)	Peroxide value (mEq O_2_/100 g oil)	Iodine value (g I_2_/100 g oil)	Saponification value (mg KOH/g oil)
A1B1	0.465 ± 0.0212^a^	1.745 ± 0.0212^a^	20.400 ± 0.1414^a^	60.800 ± 1.2728^a^	214.695 ± 2.2274^a^
A1B2	0.880 ± 0.9970^a^	0.615 ± 0.2616^b^	5.850 ± 0.3111^b^	65.550 ± 0.6364^a^	211.835 ± 0.8132^a^
A2B1	0.230 ± 0.0000^a^	0.790 ± 0.2828^b^	11.955 ± 2.8496^c^	88.300 ± 3.2527^b^	259.305 ± 42.9567^a^
A2B2	0.055 ± 0.0071^a^	0.790 ± 0.0141^b^	11.245 ± 0.9970^c^	86.500 ± 2.5456^b^	±8.4358^a^
SNI^∗^	—	< 3	< 5	< 140	—

*Note:* A1B1: crude oil extraction from trimming using the dry rendering method, A1B2: crude oil extraction from the belly using the dry rendering method, A2B1: crude oil extraction from trimming using the wet rendering method, and A2B2: crude oil extraction from the belly using the wet rendering method. Data followed by different uppercase letters indicate significantly different (*p* < 0.05).

^∗^National Standardization Agency of Indonesia [44].

**Table 3:** Common quality parameters for crude and refined fish oil.

Parameter	Typical limit	Interpretation	Reference
Free fatty acid	≤ 3.0% (crude); ≤ 1.5% (refined)	High FFA indicates hydrolytic rancidity	[54]
Peroxide value	≤ 5 mEq O_2_/kg (refined); ≤ 20 (crude)	Indicates primary oxidation products	[54]
Anisidine value	≤ 20–30	Indicates secondary oxidation (aldehydes)	[50, 55]
Iodine value	100–200	Reflects the unsaturation level of fatty acids	[56]
Saponification value	180–200 mg KOH/g (refined oils)	Indicates molecular weight of fatty acids	[53, 56]

**Table 4:** The physical, chemical, microbiological, and metal properties of crude Pangasius oil from the best treatment.

Parameters	Unit	Value	Limit of detection
Physical properties:			
Color (Lovibond Yellow 5.25^∗∗^ cell)	—	65.00	—
Color (Lovibond Red 5.25^∗∗^ cell)	—	2.90	—
Melting point	°C	33.00	—
Fatty acid profiles:			
Omega-3	mg/100 g	1007.30	—
Omega-6	mg/100 g	12,329.60	—
Omega-9	mg/100 g	36,268.55	—
Docosahexaenoic acid (DHA)	mg/100 g	173.60	—
Eicosapentaenoic acid (EPA)	mg/100 g	176.00	—
Oxidation level:			
Anisidine value (AnV)	AnV	5.88	—
Chemical properties:			
Butyric acid (C4:0)	%	—	—
Caproic acid (C6:0)	%	—	—
Caprylic acid (C8:0)	%	0.51	—
Capric acid (C10:0)	%	0.09	—
Lauric acid (C12:0)	%	2.42	—
Myristic acid (C14:0)	%	7.74	—
Palmitic acid (C16:0)	%	40.70	—
Stearic acid (C18:0)	%	9.90	—
Oleic acid (C18:1n9c)	%	21.20	—
Linoleic acid (C18:2n6cc)	%	10.60	—
Linolenic acid (C18:3)	%	6.83	—
Vitamins:			
Vitamin A	IU/g	161.65	—
Vitamin D	IU/g	192.40	—
Vitamin K	IU/g	3.20	—
Microbiologies:			
*Salmonella* sp.	25 g	Negative	—
*Escherichia coli*	MPN/g	< 3	—
Heavy metals			
Mercury (Hg)	mg/kg	Not detected	0.0008
Cadmium (Cd)	mg/kg	Not detected	0.0002
Arsenic (As)	mg/kg	Not detected	0.0004
Tin (Sn)	mg/kg	Not detected	0.005
Lead (Pb)	mg/kg	Not detected	0.0004

*Note:* The Lovibond Yellow 5.25^∗∗^ cell, mean: Lovibond Yellow testing using cell cuvette (glass tube) 5.25 inch thickness. The Lovibond Red 5.25^∗∗^ cell, mean: Lovibond Red testing using cell cuvette (glass tube) 5.25 inch thickness.

^∗∗^inch, a dimension (thickness) of cell cuvette (glass tube) used for Lovibond color testing, with a thickness of 5.25 inches.

**Table 5:** Yield of crude Pangasius oil extraction.

No.	Treatment	Maximum yield (%)	Minimum yield (%)	Average yield (%)
1	A1B1	22.92	22.50	22.71
2	A1B2	25.03	25.00	25.02
3	A2B1	14.10	14.08	14.09
4	A2B2	15.22	15.09	15.16

*Note:* A1B1: crude oil extraction from trimming using the dry rendering method, A1B2: crude oil extraction from the belly using the dry rendering method, A2B1: crude oil extraction from trimming using the wet rendering method, and A2B2: crude oil extraction from the belly using the wet rendering method.

**Table 6:** The calculation results of the value-added potentials from the crude Pangasius oil business.

No.	Variable	Value
*I*	*Output, input, and price*	
1	Output (kg)	1650.00
2	Raw material input (kg)	6600.00
3	Labor input (DWH)	1.00
4	Conversion factors (input/output)	0.25
5	Field labor coefficient (DWH/kg)	0.000152
6	Output price (U.S.$/kg)	8.95
7	Average labor wage (U.S.$/DWH)	8.83

*II*	*Profit and revenue (IDR/kg raw material)*	
8	Input price (U.S.$/kg)	0.44
9	Transaction fees (U.S.$/kg)	0.18
10	Output value (U.S.$/L)	2.24
11	Value-added (U.S.$/L) Value-added ratio (%)	1.62 72.48
12	Labor income (U.S.$/L) Labor incentives (%)	0.001338 0.0825
13	Profit (U.S.$/L) Profit rate (%)	1.62 72.42

*III*	*Production factor services (U.S.$/kg of raw materials)*	
14	Margin (U.S.$/L) Direct labor income (%) Other input donations (%) Company profits (%)	1.80 0.0744 9.85 90.08

*Note:* Source: U.S.$ = American dollar (value of 1 U.S.$ exchange rate IDR 15,955.30).

Abbreviation: DHW = daily human works.

## Data Availability

The data used to support the findings of the study are included within the article.

## References

[1] Lubis R., Bathara L., Warningsih T. (2020). Analisis Usaha Pembesaran Ikan Patin (Pangasius Sutchi) Dalam Kolam Di Desa Padang Mutung Kecamatan Kampar Kabupaten Kampar Provinsi Riau. *Jurnal Perikanan dan Kelautan*.

[2] Novianty R. (2023). Edukasi Pembuatan Minyak Ikan Patin Multifungsi. *Jurnal Pengabdian Kepada Masyarakat*.

[3] Fallo I. B., Linggi Y., Tjendanawangi A. (2023). Laju Pertumbuhan Benih Ikan Patin Siam (Pangasius Hyphopthalmus) Yang Diberi Kombinasi Pakan Pelet Dan Tepung Artemia (*Artemia salina*). *Jurnal Aquatik*.

[4] Nurilmala M., Nurhayati T., Roskananda R. (2018). Limbah Industri Filet Ikan Patin Untuk Hidrolisat Protein. *Jurnal Pengolahan Hasil Perikanan Indonesia*.

[5] Iwo A. (2019). *Potensi Produksi Minyak Ikan Dari Jeroan Ikan Patin*.

[6] Kamini P. S., Santoso J., Heri Suseno S. (2016). Ekstraksi Dry Rendering Dan Karakterisasi Minyak Ikan Dari Lemak Jeroan Hasil Samping Pengolahan Salai Patin Siam. *Jurnal Pengolahan Hasil Perikanan Indonesia*.

[7] Ayu D. F., Diharmi A., Ali A. (2019). Characterization of the Oil From the Abdomen Part of Smoked Catfish (Pangasius Hypophthalmus) Processing By-Product. *Jurnal Pengolahan Hasil Perikanan Indonesia*.

[8] Febrianto R., Sudarno S. (2020). Fish Oil Production Process From Waste Catfish (Pangasius Pangasius) in Balai Besar Pengujian Penerapan Hasil Perikanan (BBP2HP) East Jakarta. *Journal of Marine and Coastal Sciences*.

[9] Adnan S., Kresnowati M. T. A. P., Marlina Y. B., Bindar Y. (2024). Sustainable Valorisation of Freshwater Fish By-Products to Gelatin and Fish Oil by Hydrothermal Extraction. *International Journal of Food Science and Technology*.

[10] Nirmal N. P., Santivarangkna C., Rajput M. S. (2022). Valorization of Fish Byproducts: Sources to End‐Product Applications of Bioactive Protein Hydrolysate. *Comprehensive Reviews in Food Science and Food Safety*.

[11] Mat Yasin N. H., Ahmad N. A. N., Mohd Hanapi M. F. (2021). Extraction of FAME From Fish Waste by Using Modified Soxhlet Method. *IOP Conference Series: Materials Science and Engineering*.

[12] Zeleke Tilinti B., Birhanu Ayichiluhim T., Mekonnen Tura A., Duraisamy R. (2023). Extraction and Characterizations of Omega 3-Fatty Acid From Cat Fish Collected From Arba Minch Chamo Lake. *Cogent Food & Agriculture*.

[13] Mamat M. N. I. B., Rahman H. A., Razali N. S. M., Hussain S. S. S., Kasim K. F., Sofian-Seng N. S. (2025). A Review on Fish Oil Extraction From Fish By-Product as Sustainable Practices and Resource Utilization in the Fish Processing Industry. *Sains Malaysiana*.

[14] Martins M. J. J., Purnamayati L., Romadhon R. (2021). Pengaruh Suhu Wet Rendering Yang Berbeda Terhadap Karakteristik Ekstrak Kasar Minyak Ikan Lele (Clarias sp.). *agriTECH*.

[15] Dave J., Ali A. M. M., Kumar N., Nagarajan M., Kieliszek M., Bavisetty S. C. B. (2024). Investigating the Impact of Wet Rendering (Solventless Method) on PUFA-Rich Oil From Catfish (Clarias Magur) Viscera. *Open Life Sciences*.

[16] Lestari D. U., Sumardianto S., Purnamayati L. (2020). The Characteristics of Striped Catfish Oil (Pangasius Hypophthalmus) Extracted by Dry Rendering Method at Different Temperatures. *Caraka Tani: Journal of Sustainable Agriculture*.

[17] Rahman N., Hashem S., Akther S., Jothi J. S. (2023). Impact of Various Extraction Methods on Fatty Acid Profile, Physicochemical Properties, and Nutritional Quality Index of Pangus Fish Oil. *Food Science and Nutrition*.

[18] Sasongko H., Indah I., Nurrochmad A., Nugroho A. E., Rohman A., Sriwijaya U. (2025). Effects of Wet Rendering Extraction on the Fatty Acid and Physicochemicalprofiles of Catfish (Pangasius Micronema Blkr.), Milkfish (Chanos chanosForsskal.) and Snakehead Fish (Chana Striata Bloch). *Food Research*.

[19] Hayami Y., Kawagoe T., Morooka Y., Siregar M. (1987). *Agricultural Marketing and Processing in the Java Highlands: Perspectives From Sunda Village*.

[20] Sumantri B., Purwoko A., Sriyoto S., Sukiyono K., Sumartono E. (2018). Economic Value A Dried Fish Business Development in Bengkulu City. *Indonesian Journal of Agricultural Research*.

[21] Ramadhan M. F., Junianto (2021). Analysis of Added Value of Fish Drumstick at Home Industry “Adisyafidz Barokah” Nagreg, Bandung Regency, West Java. *Asian Journal of Fisheries and Aquatic Research*.

[22] Nabilasari M., Sumantri B., Sriyoto S. (2022). Value-Added Analysis of the Dried Fish Manufacturing Industry in Bengkulu City. *Journal of Global Sustainable Agriculture*.

[23] Alif R. H. (2023). Determinants of Economic Value Addition of Industrial Tuna Fish Processors in the Sea Food Processing Sub-Chain in Malaysia. *Indian Journal of Economics and Business*.

[24] Amri U., Diharmi A., Sukmiwati M. (2021). Characteristics of Catfish Oil, Red Palm Oil and Shark Liver Oil as Functional Foods. *Depik*.

[25] Fortuna Ayu D., Sihombing A. T. E., Diharmi A. (2022). Pemurnian Minyak Ikan Patin Menggunakan Magnesol Dalam Pembuatan Mayones. *Jurnal Pengolahan Hasil Perikanan Indonesia*.

[26] AOAC International (2016). Official Methods of Analysis of AOAC International. *Journal of the Association of the Official Analytical Chemists*.

[27] Ayeloja A. A., Jimoh W. A., Garuba A. O. (2024). Nutritional Quality of Fish Oil Extracted From Selected Freshwater Fish Species. *Food Chemistry Advances*.

[28] International Organization for Standardization (2017). *Animal and Vegetable Fats and Oils: Gas Chromatography of Fatty Acid Methyl Esters: Part 2: Preparation of Methyl Esters of Fatty Acids*.

[29] National Standardization Agency of Indonesia (2015). *SNI ISO 3961:2015-Lemak Dan Minyak Hewani Dan Nabati-Penentuan Bilangan Iod (ISO 3961:2013, IDT) (Animal and Vegetable Fats and Oils-Determination of Iodine Number)*.

[30] National Standardization Agency of Indonesia (2006). *SNI 01-2332.2-2006. Cara Uji Mikrobiologi-Bagian 2: Penentuan Salmonella Pada Produk Perikanan (Microbiological Test Methods-Part 2: Determination of Salmonella in Fishery Products)*.

[31] National Standardization Agency of Indonesia SNI 2332.1:2015. Microbiological Test Methods-Part 1: Determination of Coliforms and Escherichia coli in Fishery Products; and Chemical Test Methods. *Part 23: Determination of Heavy Metals: Mercury (Hg), Lead (Pb), Cadmium (Cd), Arsenic (As) and Tin (Sn) in Fishery Products Using Inductively Coupled Plasma Mass Spectrometer (ICP-MS) Simultaneously*.

[32] AOCS (2017). Official Method CC 13b-45: Color of Fats and Oils (Lovibond Tintometer Method, Visual Measurement in 5.25-Inch Cell). *Official Methods and Recommended Practices of the AOCS*.

[33] International Organization for Standardization (ISO) (2021). *ISO 6321:2021—Animal and Vegetable Fats and Oils—Determination of Melting Point in Open Capillary Tubes (Slip Point)*.

[34] International Organization for Standardization (ISO) (2016). *ISO 6885:2016—Animal and Vegetable Fats and Oils—Determination of Anisidine Value*.

[35] Latimer G. W., AOAC International (2019). Official Method 2001.13: Vitamin A (Retinol) in Foods—Liquid Chromatography (First Action 2001). *Official Methods of Analysis of AOAC International*.

[36] European Committee for Standardization (Cen) (2009). *EN 12821:2009. Foodstuffs–Determination of Vitamin D by High Performance Liquid Chromatography–Measurement of Cholecalciferol (D3) or Ergocalciferol (D2)*.

[37] Latimer G. W., AOAC International (2019). Official Method 999.15: Vitamin K_1_ (Phylloquinone) in Milk and Infant Formula—Liquid Chromatography With Post-Column Reduction and Fluorescence Detection (Final Action). *Official Methods of Analysis of AOAC International*.

[38] Rahmawati A. S., Erina R. (2020). Rancangan Acak Lengkap (RAL) Dengan Uji Anova Dua Jalur. *OPTIKA: Jurnal Pendidikan Fisika*.

[39] Sikone H. Y., Hartono B., Suyadi, Utami H. D., Nugroho B. A. (2022). Value-Added Analysis of the Meat Agroindustry in Indonesia. *Online Journal of Animal and Feed Research*.

[40] Honold P. J., Nouard M. L., Jacobsen C. (2016). Fish Oil Extracted From Fish‐Fillet By‐Products is Weakly Linked to the Extraction Temperatures But Strongly Linked to the Omega‐3 Content of the Raw Material. *European Journal of Lipid Science and Technology*.

[41] Indah I., Rohman A., Lestari L. A. (2022). Physicochemical Characterization Patin Fish Oil (Pangasius Micronema) is Refined Using Bentonite and Activated Carbon. *Journal of Food and Pharmaceutical Sciences*.

[42] Carneiro C. R., Alhaji A. M., da Silva C. A. S., de Sousa R. d. C. S., Monteiro S., Coimbra J. S. D. R. (2023). Potential Challenges of the Extraction of Carotenoids and Fatty Acids From Pequi (Caryocar Brasiliense) Oil. *Foods*.

[43] Miranda P. H. S., Santos A. C. D., Freitas B. C. B. D., Martins G. A. D. S., Vilas Boas E. V. D. B., Damiani C. (2021). A Scientific Approach to Extraction Methods and Stability of Pigments From Amazonian Fruits. *Trends in Food Science & Technology*.

[44] National Standardization Agency of Indonesia (2018). *SNI Minyak Ikan Murni (Refined Fish Oil)–Syarat Mutu Dan Pengolahan (SNI, Refined Fish Oil)–Quality and Processing Requirements*.

[45] Suseno S. H., Rizkon A. K., Jacoeb A. M., Kamini, Listiana D. (2021). Fish Oil Extraction as a By-Product of Tilapia (Oreochromis sp.) Fish Processing With Dry Rendering Method. *IOP Conference Series: Earth and Environmental Science*.

[46] Mohanarangan A. B. *Extraction of Omega-3 Fatty Acids From Atlantic Herring (Clupea Harengus)*.

[47] Nurbayasari R., Bandol Utomo B. S., Basmal J., Hastarini E. (2017). Pemurnian Minyak Ikan Patin Dari Hasil Samping Pengasapan Ikan. *Jurnal Pascapanen dan Bioteknologi Kelautan dan Perikanan*.

[48] Aminah S. (2010). Bilangan Peroksida Minyak Goreng Curah Dan Sifat Organoleptik Tempe Pada Pengulangan Penggorengan. *Jurnal Gizi dan Pangan*.

[49] Chantachum S., Benjakul S., Sriwirat N. (2000). Separation and Quality of Fish Oil From Precooked and Non-Precooked Tuna Heads. *Food Chemistry*.

[50] Shahidi F., Ambigaipalan P. (2018). Omega-3 Polyunsaturated Fatty Acids and Their Health Benefits. *Annual Review of Food Science and Technology*.

[51] Song G., Dai Z., Shen Q., Peng X., Zhang M. (2018). Analysis of the Changes in Volatile Compound and Fatty Acid Profiles of Fish Oil in Chemical Refining Process. *European Journal of Lipid Science and Technology*.

[52] Asoiro F. U., Okonkwo W. I., Akubuo C. O., Nweze N. O., Anyanwu C. N. (2019). Effects of Temperature and Time on Oil Extraction From Some Nigerian Indigenous Fresh Water Microalgae Species. *Agricultural Engineering International: CIGR Journal*.

[53] Humadi J. I., Jafar S. A., Ali N. S. (2023). Recovery of Fuel From Real Waste Oily Sludge Via a New Eco-Friendly Surfactant Material Used in a Digital Baffle Batch Extraction Unit. *Scientific Reports*.

[54] Codex Alimentarius Commission (2017). *Standard for Fish Oil CXS 329-2017*.

[55] [GOED] Global Organisation for EPA and DHA (2015). *Oxidation in Omega-3 Oils: An Overview*.

[56] Irnawati I., Nadia L. O. M. H., Windarsih A. (2023). Fatty Acid Composition, Biological Activity and Authentication of Marine Fish Oil. *Food Research*.

[57] Šimat V. (2021). Valorization of Seafood Processing By-Products. *Valorization of Agri-Food Wastes and By-Products*.

[58] Widowati D., Siswanti, Anandito R. B. K., Purnamayati L. (2024). The Changes in the Physical and Chemical Quality of Catfish Oil (*Pangasius sp.*) With Rice Bran Oil Addition During Room Temperature Storage. *Food Research*.

[59] IFOMA (1998). Quality and Standards for Fish Oils. *International Fishmeal and Fish Oil Organisation-INFORM*.

[60] Reski S., Mundhofir F. E. P., Murbawani E. A. (2021). Efficacy of Catfish (Pangasius Hypophthalmus) Oil to Overcome Stunting by Reducing Inflammatory Condition. *International Journal of Pharmacy and Pharmaceutical Sciences*.

[61] Lubis et al. H. F., Idrus E., Sastradipura D. F. S., Suseno S. H. (2024). Characteristics and Nutrition of Fat-Soluble Vitamins From the Patin Fish Oil (Pangasius Hypothalmus) and Their Potential in Dental Health. *Egyptian Journal of Aquatic Biology and Fisheries*.

[62] Oterhals A., Berntssen M. H. G. (2010). Effects of Refining and Removal of Persistent Organic Pollutants by Short-Path Distillation on Nutritional Quality and Oxidative Stability of Fish Oil. *Journal of Agricultural and Food Chemistry*.

[63] Setiadi S., Nurmalina R., Suharno S. (2018). Analisis Kinerja Rantai Pasok Ikan Nila pada Bandar Sriandoyo di Kecamatan Tugumulyo Kabupaten Musi Rawas. *Mix Scientific Journal of Management*.

[64] Sumardi A., Rira D., Damang K., Munizu M. (2017). Determinant Factors of Supply Chain Performance: Case at Seaweed Business in Takalar Regency, South Sulawesi Province of Indonesia. *International Journal of Economic Research*.

